# Dynamical control of nanoscale light-matter interactions in low-dimensional quantum materials

**DOI:** 10.1038/s41377-024-01380-x

**Published:** 2024-01-25

**Authors:** Yeonjeong Koo, Taeyoung Moon, Mingu Kang, Huitae Joo, Changjoo Lee, Hyeongwoo Lee, Vasily Kravtsov, Kyoung-Duck Park

**Affiliations:** 1 Department of Physics, Pohang University of Science and Technology (POSTECH), Pohang, 37673 Republic of Korea; 2 School of Physics and Engineering, ITMO University, Saint Petersburg, 197101 Russia

**Keywords:** Optical metrology, Nanophotonics and plasmonics

## Abstract

Tip-enhanced nano-spectroscopy and -imaging have significantly advanced our understanding of low-dimensional quantum materials and their interactions with light, providing a rich insight into the underlying physics at their natural length scale. Recently, various functionalities of the plasmonic tip expand the capabilities of the nanoscopy, enabling dynamic manipulation of light-matter interactions at the nanoscale. In this review, we focus on a new paradigm of the nanoscopy, shifting from the conventional role of imaging and spectroscopy to the dynamical control approach of the tip-induced light-matter interactions. We present three different approaches of tip-induced control of light-matter interactions, such as cavity-gap control, pressure control, and near-field polarization control. Specifically, we discuss the nanoscale modifications of radiative emissions for various emitters from weak to strong coupling regime, achieved by the precise engineering of the cavity-gap. Furthermore, we introduce recent works on light-matter interactions controlled by tip-pressure and near-field polarization, especially tunability of the bandgap, crystal structure, photoluminescence quantum yield, exciton density, and energy transfer in a wide range of quantum materials. We envision that this comprehensive review not only contributes to a deeper understanding of the physics of nanoscale light-matter interactions but also offers a valuable resource to nanophotonics, plasmonics, and materials science for future technological advancements.

## Abstract (web summary)

We discuss the recent progress on the dynamic control of tip-induced light-matter interactions, especially for the low dimensional quantum materials. This review is divided into three parts based on the type of tip-induced control, i.e., gap control of tip-cavity, tip-pressure control, and near-field polarization control.

## Graphical abstract

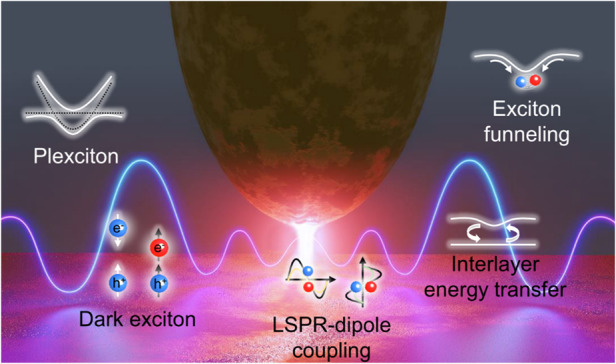

## Introduction

Low-dimensional quantum materials, such as two-dimensional (2D) transition metal dichalcogenides (TMDs), semiconductor nanowires, and quantum dots (QDs) have garnered significant attention due to their exceptional optical, electrical, and structural properties^1–4^. Their unique properties are inherited from the reduced dimensionality, which leads to the new physical phenomena, such as the increased quantum confinement effect^5^ and decreased dielectric screening effects^6^. These characteristics exhibit remarkable sensitivity to external engineering strategies, offering wide tunability that distinguishes them from their bulk counterparts^7,8^. By leveraging the tunability, we can precisely control their optical response^9,10^, electrical conductivity^11,12^, and structural characteristics^13,14^, rendering them promising candidates for a multitude of applications in nanophotonics, optoelectronics, and beyond.

Since the length scale of the interesting phenomena reduces down to the nanoscale, the intriguing physical processes of low-dimensional quantum materials cannot be investigated by the conventional microscale spatial imaging methods. Nanoscopy approaches, such as scanning probe microscopy (SPM)^15–19^ and tip-enhanced spectroscopy^20–24^, have enabled us to directly visualize the nanoscale characteristics of the low-dimensional quantum materials with extraordinary accessibility. Using the plasmonic tip, nanoscopy allows for the spatially resolved characterizations of the electronic structure, excitonic properties, and local optical responses of the materials. This capability has revealed a wealth of interesting physical phenomena at the nanoscale, including exciton dynamics, quantum confinement effects, and the interplay between electronic and optical properties. For instance, the ability to probe the optically dark states, which are typically challenging to access, opens up possibilities for the manipulation of light emission and tailored photon sources^25–27^. Nanoscopy also unveils the drift-dominant exciton funneling facilitated by the nanoscale strain, providing insights into strain engineering for improved optoelectronic devices^28–30^. Moreover, achieving strong coupling with nanoscale mode volumes allows for the exploration of cavity quantum electrodynamics effects, enabling precise control over light-matter interactions at the fundamental level^31–33^.

**Fig. 1:**
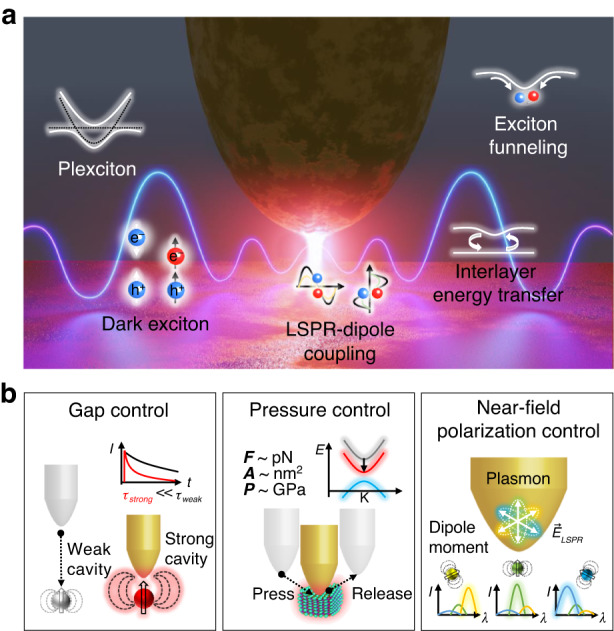
**Illustration of dynamic tip-induced control of light-matter interactions. a** Unique properties of low-dimensional quantum materials that can be dynamically manipulated by the tip-induced control. **b** Schematic illustrations for the three types of tip-induced control. Gap control for engineering the cavity-emitter coupling strength, pressure control for engineering the strain, bandgap, and emission energy of quantum materials, and near-field polarization control for engineering the plasmon-emitter coupling with high selectivity

However, a plethora of physics captured by the conventional nanoscopy is just the tip of the iceberg, whereas the complex interplay between multiple degrees of freedom, such as electronic, vibrational, and photonic modes, as well as their coupling dynamics remain largely unexplored. Therefore, there is a growing demand for innovative approaches to actively manipulate the light-matter interactions. In this review, we discuss the recent progress on the dynamic control of tip-induced light-matter interactions, especially for the low-dimensional quantum materials. As illustrated in Fig. 1b, we divide the main text into three parts based on the type of tip-induced control, i.e., gap control of tip-cavity, tip-pressure control, and near-field polarization control. For low-dimensional semiconductors, such as 0D QDs and 2D TMDs, we discuss the modulation of radiative emission depending on the variation of tip-sample distance (part 1) and the modified strain and band gap properties induced by the GPa scale tip-pressure (part 2). Additionally, we discuss the near-field polarization control at the tip apex achieved by the geometrical tip modification and adaptive wavefront shaping of the excitation beam (part 3). We believe that this review will guide the future direction of manipulation of the quantum light-matter interaction by introducing a new paradigm of near-field microscopy and suggesting the recently developed approaches to discover the hidden nature of the emerging quantum materials.

### Tip-induced control of radiative emissions via cavity-gap engineering

Many types of light-matter interaction play a crucial role in photonic and optoelectronic device applications, e.g., lasers, light-emitting diodes (LEDs), single photon emitters, photodiodes, and solar cells^34–38^. Since plasmonic cavities can modify light-matter interactions with the nanoscale mode volume, cavity-quantum electrodynamics (cavity-QED) study with many different platforms, such as nanoparticles on mirror (NPoM)^39–44^, bowtie antennas^45–51^, and nanogap structures^52–58^, has become ever more important. However, the static geometry of the conventional plasmonic cavities highly restricts the dynamic controllability of light-matter interactions, e.g., Purcell enhancement^48,59–66^, radiative decay rate^57,67–74^, and coupling strength^33,42,43,47,49,75–80^, due to their fixed cavity mode volume. Hence, to achieve the desired nanoscale modulation of the cavity-gap in a reversible manner, the plasmonic tip-cavity approach combined with a SPM has been demonstrated.

**Fig. 2:**
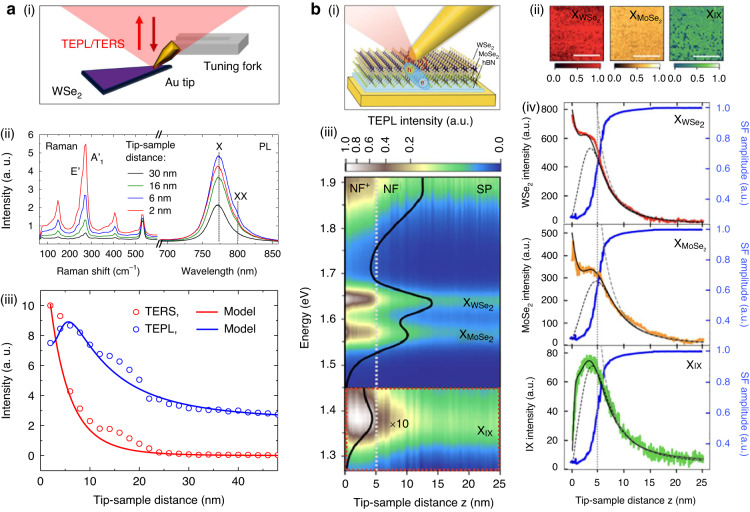
**Tip-induced control of photoluminescence and Raman scattering via cavity-gap engineering. a** Manipulation of the TERS/TEPL response of a WSe_2_ monolayer. (i) Schematic of the tip-enhanced nano-spectroscopy. (ii) TERS/TEPL spectra at the selected typical tip-sample distances. (iii) Evolution of the TERS/TEPL intensity of a WSe_2_ monolayer as the tip-sample distance decreases, fitted with the rate equation model. **b** Purcell effect-induced TEPL manipulation of a TMD heterostructure. (i) Schematic of tip-enhanced nanocavity clock spectroscopy. (ii) TEPL images of the heterobilayer for the integrated intensity of ${{\rm{X}}}_{{\rm{WS}}{{\rm{e}}}_{2}}$ (red), ${{\rm{X}}}_{{\rm{MoS}}{{\rm{e}}}_{2}}$ (yellow), and ${{\rm{X}}}_{{\rm{IX}}}$ (green). Scale bar is 500 nm. (iii) Evolution of TEPL spectra with respect to the tip-sample distance $z$. (iv) Distance-dependent PL intensity of ${{\rm{X}}}_{{\rm{WS}}{{\rm{e}}}_{2}}$, ${{\rm{X}}}_{{\rm{MoS}}{{\rm{e}}}_{2}}$, and ${{\rm{X}}}_{{\rm{IX}}}$, fitted with the coupled rate equation model. **a** Reproduced with permission^81^. Copyright [2016] American Chemical Society. **b** Reproduced with permission^86^. Copyright [2021] American Chemical Society

Figure 2a-(i) shows the schematic illustration of the tip-enhanced Raman scattering (TERS) and photoluminescence (TEPL) spectroscopy based on the shear-force atomic force microscopy (AFM) to investigate the tip-induced optical responses of a TMD monolayer^81^. When the Au tip closely approaches the emitters, the optical excitation rate (${\Gamma }_{{\rm{e}}}$) is enhanced due to the field localization effect, which promotes the interaction of the light with the phonon and exciton. The distance-dependent excitation rate is well-described with an equation ${\Gamma }_{{\rm{e}}}\left(z\right)\propto {\left(1/\left(R+z\right)\right)}^{4}$ with the tip apex radius $R$ and tip-sample distance $z$. In this condition, TERS and TEPL signals are additionally enhanced by different mechanisms. In the case of TERS, the charge-transfer resonance between the TMD monolayer and the metal tip enhances the Raman scattering^82^. For TEPL, the spontaneous emission rate is increased by the Purcell effect with the well-known Purcell factor ${F}_{{\rm{p}}}\propto Q/V$, where $Q$ and $V$ are the quality factor of an emitter and the mode volume of a cavity. Figure 2a-(ii) shows the distance-dependent TERS/TEPL spectra of a WSe_2_ monolayer at the selected distances between the tip and sample. By modulating the tip-sample distance, the intensity of the TERS/TEPL can be adjusted to the desired level. Furthermore, the experimental data of the TERS/TEPL intensity with respect to the tip-sample distance shows a good agreement with the simulation data, as shown in Fig. 2a-(iii). The gradual enhancement of the TERS/TEPL intensity with the decreasing tip-sample distance at $d$ < 20 nm can be explained by the rate equation for excited state population of the phonons and excitons coupled with the tip-plasmon. However, at the distance $d$ < 5 nm, the PL intensity is decreased due to the near-field polarization transfer between the WSe_2_ exciton and the metal tip causing nonradiative damping and PL quenching. PL quenching is a classical phenomenon that occurs due to near-field energy transfer from a semiconductor to a metal, leading to nonradiative decay. In addition, a transition from the classical regime to the quantum regime occurs at smaller gaps, primarily driven by nonlocal screening and charge tunneling effects, as elucidated in previous research^83^. Nonlocal screening pertains to the behavior of electrons in a metal and how they interact with screen charges at the metal-semiconductor gap. This phenomenon can cause deviations in the spatial distribution of electron gas within the metal structure compared to the geometric boundaries, resulting in a decrease of TEPL intensity at smaller gaps^84,85^. Quantum tunneling, on the other hand, is a well-known electron behavior in sub-nanometer gaps, contributing to nonradiative decay of excitons in the semiconductor via charge transfer to the metallic tip in TEPL experiments. The distance-dependent change in TEPL from the classical to the quantum regime can be effectively described by modified rate equation models, as evidenced in prior studies^81,84^. This study demonstrated the near-field light-matter interactions in a TMD monolayer with TEPL and TERS, revealing the potential of tip-induced cavity-gap engineering for modulating nano-optical properties of emerging 2D materials.

The similar tip-induced approach was also employed to control the light-matter interactions in a TMD heterobilayer, especially by manipulating competing TEPL responses of intra- and inter-layer excitons. As shown in Fig. 2b, May et al.^86^ investigated TEPL properties of the WSe_2_/MoSe_2_ heterostructure. First of all, the spatially homogeneous region over a 1 μm^2^ was confirmed through the TEPL imaging (Fig. 2b-(ii)). Then, the authors were able to manipulate the TEPL spectra of intralayer excitons (${{\rm{X}}}_{{\rm{WS}}{{\rm{e}}}_{2}}$ and ${{\rm{X}}}_{{\rm{MoS}}{{\rm{e}}}_{2}}$), interlayer exciton (${{\rm{X}}}_{{\rm{IX}}}$), and surface plasmon (SP) as a function of the tip-sample distance $z$ from 25 nm to 0 nm, as shown in Fig. 2b-(iii). From the measurement results, the authors could classify the near-field responses of the heterobilayer into three ranges depending on $z$: (1) a range of near-field (NF) ($z$ ≥ 5 nm), (2) a range of suppressed enhancement (1 nm < $z$ < 5 nm), and (3) a NF^+^ range ($z$ ≤ 1 nm). In the NF range, PL intensity of ${{\rm{X}}}_{{\rm{WS}}{{\rm{e}}}_{2}}$, ${{\rm{X}}}_{{\rm{MoS}}{{\rm{e}}}_{2}}$, and ${{\rm{X}}}_{{\rm{IX}}}$ is continuously enhanced as the increasing excitation rate dominates over PL quenching in the proximity of the Au tip. In the range of 1 nm < $z$ < 5 nm, the PL quenching has a predominance of the near-field response over the increased excitation rate via dipole coupling of the Au tip and ultrafast ohmic Drude damping. By contrast, the strong Purcell effect enhances the spontaneous emission rate in the range of $z$ ≤ 1 nm (NF^+^ region). Interestingly, in this region, only the TEPL of intralayer excitons is largely increased due to Purcell enhancement. Concurrently, the ${{\rm{X}}}_{{\rm{IX}}}$ are quenched, because the tip-enhanced nonradiative damping of ${{\rm{X}}}_{{\rm{IX}}}$ becomes much faster than the intralayer charge transfer. This complex competing spectral behaviors of ${{\rm{X}}}_{{\rm{IX}}}$, ${{\rm{X}}}_{{\rm{WS}}{{\rm{e}}}_{2}}$, and ${{\rm{X}}}_{{\rm{MoS}}{{\rm{e}}}_{2}}$ with respect to the decreasing $z$ is modeled by the coupled rate equations, as shown in Fig. 2b-(iv). This work presents a method for controlling the radiative emission of complex quantum systems solely by dynamically modulating the tip-sample distance. Moreover, the proposed method can be applied to investigate the dynamics of excitonic systems in a wide range of materials.

**Fig. 3:**
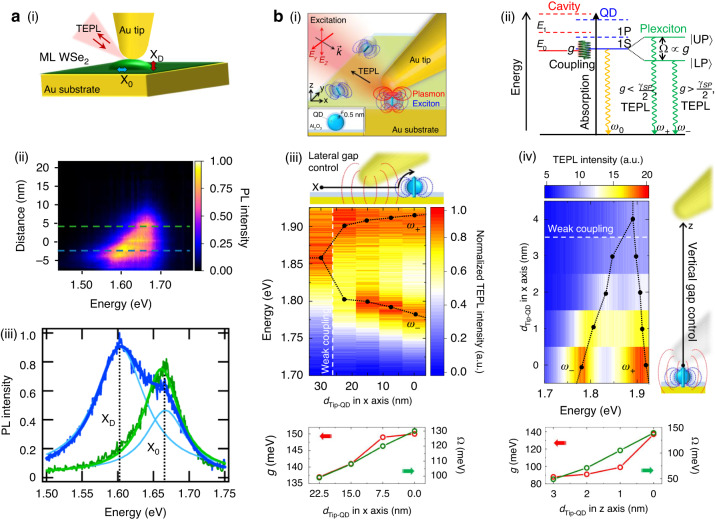
**Tip-induced control of radiative emission of dark exciton and strong coupling of quantum dot exciton via cavity-gap engineering. a** Probing radiative emission of the dark exciton (${{\rm{X}}}_{{\rm{D}}}$) in a WSe_2_ monolayer via TEPL. (i) Schematic of the TEPL to probe and control ${{\rm{X}}}_{{\rm{D}}}$ in a TMD nanobubble. (ii) Continuous plot of TEPL spectra with respect to the tip-sample distance. (iii) TEPL spectra at different tip-sample distances indicated by dotted lines in (**a**-(ii)). **b** Control of the strong coupling between the tip-cavity and the single quantum emitter. (i) Schematic of the TESC spectroscopy. (ii) Energy diagram of the tip-cavity, QD, and the polariton state of plasmon and exciton, i.e., plexciton. Modified TEPL spectra depending on the lateral (iii) and the vertical (iv) tip-sample distance change. **a** Reproduced with permission^26^. Copyright [2023] American Chemical Society. **b** Reprinted with permission^31^. Copyright © 2019 Park et al., some rights reserved; exclusive licensee American Association for the Advancement of Science. No claim to original U.S. Government Works. Distributed under a Creative Commons Attribution NonCommercial License 4.0 (CC BY-NC)

In addition to the bright excitons, the strong out-of-plane optical field at the tip-cavity can reveal the forbidden optical states in 2D TMDs. Park et al.^25^ demonstrated the first work of tip-induced probing and controlling the room temperature dark exciton (${{\rm{X}}}_{{\rm{D}}}$) radiation in a WSe_2_ monolayer, which typically exhibits the spin-forbidden nonradiative decay with the intrinsic out-of-plane oriented transition dipole moment. Following that, the recent study of Hasz et al.^26^ showed the tip-induced ${{\rm{X}}}_{{\rm{D}}}$ radiation in nano-bubbles of a WSe_2_ monolayer (Fig. 3a-(i)). The physical mechanism of the tip-induced radiative control of the ${{\rm{X}}}_{{\rm{D}}}$ is divided into two steps as follows. First, the linearly polarized incident light is strongly confined in the tip-cavity formed with the metal substrate resulting in the large enhancement of the out-of-plane optical field due to the dipole-dipole interaction. The confined light then effectively excites the vertically oriented ${{\rm{X}}}_{{\rm{D}}}$ in the WSe_2_ monolayer. Additionally, the spontaneous emission rate of the ${{\rm{X}}}_{{\rm{D}}}$ is increased by the Purcell effect, which, in general, gives maximum enhancement when the orientation of the emitter aligns with the polarization axis of the localized fields. Therefore, the Purcell effect of ${{\rm{X}}}_{{\rm{D}}}$ in the tip-cavity achieved an extraordinarily large enhancement of the radiative emission rate. In addition, as illustrated in Fig. 3a-(i), a side-illumination TEPL geometry is particularly advantageous for the effective collection of dark exciton emissions, which possess a *k*-vector parallel to the 2D surface. Note that the lower bandgap energy of ${{\rm{X}}}_{{\rm{D}}}$ compared to X_0_ can be attributed to the spin-forbidden transition to the lower-energy conduction band. The degree of confinement of the incident light and the enhancement of the radiative emission rate sensitively depends on the cavity-gap, as described previously. Therefore, the radiative emission rate of X_D_ is controlled by modulating the tip-sample distance, as shown in Fig. 3a-(ii). Figure 3a-(iii) shows the two extracted TEPL spectra from Fig. 3a-(ii) at different distances of the tip and sample. For the 10 nm of tip-sample distance (green), only the bright exciton emission is observed. By contrast, as the tip-sample distance is decreased, ${{\rm{X}}}_{{\rm{D}}}$ emission is observed, while the intensity of the bright exciton decreases due to dipole coupling and energy transfer with Drude relaxation in the metal substrate and tip. Therefore, the forbidden optical state in conventional light-matter coupling was allowed to be brightened via nanoscale engineering of the tip-cavity gap. Through the dynamical engineering of radiative emission of ${{\rm{X}}}_{{\rm{D}}}$ at room temperature, we envision many different types of device applications in quantum nano-optoelectronics, utilizing its long radiative lifetime and coherence time. Since these studies were light-matter interaction phenomena in the weak coupling regime, one could only observe modifications in optical intensity induced by the coupling of plasmon with excitons and phonons. In the strong coupling regime, on the other hand, the modifications in photon energy can also be pronouncedly observed in addition to the tip-enhanced optical responses. However, because of its dominant out-of-plane optical field in the cavity-gap, tip-cavity is less advantageous for the strong coupling with in-plane excitons in 2D materials. While metasurfaces have been suggested for achieving strong coupling with in- plane excitons^87^, tip-cavity has stood out as a suitable platform for the out-of-plane dipole emitter, such as QD. Park et al.^31^ introduced a concept of tip-enhanced strong coupling (TESC) spectroscopy, by forming a nanoscale tip-cavity for a single emitter. In this study, a single CdSe/ZnS QD was placed between the Au tip and the Au surface, as illustrated in Fig. 3b-(i). Since this Au-Au junction provides a nanoscale mode volume in the tip-cavity, it can induce strong coupling between the cavity plasmon and the QD exciton. To induce the strong coupling state, the transition dipole moment of a QD should be also oriented vertically (along the tip-cavity axis) because the coupling strength ($g$) depends on the dipole orientation of the excitons ($\vec{\mu }$) and plasmon polarization (${\vec{E}}_{0}$)^88^:

1 $${{\hslash }}g\propto \vec{\mu }\cdot \vec{{E}_{0}}\propto \vec{\mu }\cdot {\vec{u}}_{{\rm{cav}}}\sqrt{\frac{N{{\hslash }}{{{\omega }}}_{{\rm{exc}}}}{{{{\epsilon }}}_{0}V}}$$

where ${\vec{u}}_{{\rm{cav}}}$ is the unit vector of the plasmon polarization of the cavity, $N$ is the number of molecules, ${\epsilon }_{0}$ is the vacuum permittivity, ${{\hslash}} {\omega }_{{\rm{exc}}}$ is the exciton energy, and $V$ is the cavity mode volume. As shown in the energy diagram of Fig. 3b-(ii), when the coupling strength is larger than the loss of the coupled system, one can facilitate the plexciton state, i.e., strong coupling state of plasmon and exciton, which shows a signature of Rabi splitting in TEPL spectra. The beauty of TESC spectroscopy compared to strong coupling studies using static plasmonic cavities^33,42,43,47,49,75–80^ is dynamic controllability of the strong coupling state. The authors could control the coupling strength of plexcitons by modulating the optical mode volume $V$ through the cavity-gap engineering in a few nm scales (${\rm{g}}\propto 1/\sqrt{{\rm{V}}}$) because the Au tip can move laterally or vertically by the AFM control. TEPL spectra in Fig. 3b-(iii), (iv) demonstrate the continuous modifications in coupling strength for the plexciton state, when the tip-cavity mode volume gradually changes. For the single isolated CdSe/ZnS QD in the TESC spectroscopy, the coupling strength of ~140 meV was observed at room temperature. The coupling strength is derived by using the coupled harmonic oscillator model with Weisskopf-Wigner approximation^89^:

2 $${I}_{{\rm{PL}}}\left({\rm{\omega }}\right)=\frac{{{\rm{\gamma }}}_{{\rm{QD}}}}{2{\rm{\pi }}}{\left|\frac{{{\rm{\gamma }}}_{{\rm{SP}}}/2-i\left({\rm{\omega }}-{{\rm{\omega }}}_{{\rm{SP}}}\right)}{\left({{\rm{\gamma }}}_{{\rm{SP}}}+{{\rm{\gamma }}}_{{\rm{QD}}}\right)/4-i\left({{\rm{\omega }}}_{{\rm{QD}}}-{{\rm{\omega }}}_{{\rm{SP}}}\right)/2-i{\left({\rm{\omega }}-{{\rm{\omega }}}_{{\rm{QD}}}\right)}^{2}+{\Omega }^{2}}\right|}^{2}$$

3 $$g=2\sqrt{{\Omega }^{2}-\frac{{\left({{\rm{\omega }}}_{{\rm{QD}}}-{{\rm{\omega }}}_{{\rm{SP}}}\right)}^{2}}{4}+\frac{{\left({{\rm{\gamma }}}_{{\rm{SP}}}-{{\rm{\gamma }}}_{{\rm{QD}}}\right)}^{2}}{16}}$$

where $\Omega$, ${\omega }_{{\rm{SP}}}$, ${\gamma }_{{\rm{QD}}}$, and ${\gamma }_{{\rm{SP}}}$ denote the vacuum Rabi frequency, the resonance frequency of QD, the resonance frequency of cavity, the decay rate of QD, and the decay rate of cavity, respectively.

This work presented a modulation of coupling strength through plasmonic cavity-gap engineering allowing for the control of the Rabi splitting in a strong coupling regime. The other advantage of TESC compared to conventional static cavities is the ability to perform a control experiment. That is, one can induce tip-enhanced strong coupling for many different emitters with the 3D movable tip-cavity, which enables control experiments in the strong coupling regime. Hence, this approach enables the tuning of quantum-optical interfaces and provides a large degree of functionality to control quantum dynamics, which can lead to the advanced development of quantum computing. Furthermore, quantum plasmonic effects in sub-nm gaps should be briefly introduced. As the cavity-gap decreases down to the quantum tunneling regime, quantum plasmonic effects play a pivotal role in light-matter interactions, leading to unique and often non-classical phenomena under this extreme cavity condition^83^. For example, a transition from plasmonic enhancement to electron tunneling was observed in a tip-cavity. In an insightful study by Kravtsov et al.^84^, the authors measured luminescence signals using a gold tip positioned above a flat gold surface while changing the tip-sample gap. Their observations revealed that the peak luminescence intensity was attained when the tip was at an approximate gap distance of $d$ ~ 1.5 nm. Notably, as the gap width reduced, PL quenching became evident, and concurrently, the luminescence spectral peaks exhibited a blue-shift, signaling the onset of electron tunneling. In addition, Zhe et al. demonstrated exciton-to-trion conversion in a TMD monolayer by using the electron tunneling regime of tip-cavity^90^. Their experimental observations underscore the fundamental distinctions between classical and quantum plasmonic regimes in the study of light-matter interactions. Classical TEPL facilitates nano-spectroscopy through plasmonic near-field enhancement but provides limited capacity to locally manipulate the excitons in 2D semiconductors. In contrast, the quantum plasmonic TEPL technique offers novel control mechanisms alongside classical nano-spectroscopy capabilities, thereby unlocking fresh avenues for concurrent nano-imaging and control within the quantum domain. Therefore, considering the unique quantum plasmonic behaviors of the sub-nm gap tip-cavity will open up exciting possibilities for investigating phenomena that were previously inaccessible. Moreover, in the quantum regime, the tip-induced approach can be used to investigate the tunneling effect in biomolecules^91,92^. However, embedding biomolecules in the gap between the tip and substrate is not an easy process, particularly in achieving reproducibility and stability. Recently, hyperspectral TERS imaging of single molecules at room temperature has been made possible by improved reproducibility via a freeze-frame approach using a thin Al_2_O_3_ capping layer^93^, yet dynamic control of light-biomolecule interactions remains challenge. Conversely, the use of static nanogap structures, coupled with the application of dynamic control factors like external electric and optical fields, offers distinct advantages, compared to tip-induced approach, when it comes to probing quantum effects within the tunneling region of biomolecules^94,95^. These recent studies are compelling examples demonstrating how this approach provides a new avenue for investigating the intricate interplay between biological molecules and light-matter interactions, ultimately leading to a deeper comprehension of these complex phenomena.

In summary, the recently demonstrated works on tip-induced control of radiative emissions via cavity-gap engineering provide a new direction for the cavity-QED studies. In contrast to the static plasmonic cavity, the dynamically modulating mode volume in the tip-cavity enables a systematic manipulation of the plasmon-coupled optical properties for various emitters, such as spontaneous emission rate, energy transfer, coupling strength, and so forth. Specifically, strong coupling significantly modifies the chemical reaction rate of molecules^96^, emission rate of single quantum dot^97^, and emission properties of 2D TMDs^42^. Therefore, we envision that the tunability of tip-cavity for a range of light-matter interactions at the nanoscale provides a new strategy for the next-generation nano-photonic device applications, overcoming the current performance limits of optoelectronic devices.

### Tip-induced control of excitons via GPa-scale pressure engineering

Strain engineering has emerged as a versatile method to modify the physical properties of materials, expanding their potential applications in various nano-optoelectronic devices^98–100^. Although several techniques, such as thermal annealing^101,102^, hydrostatic pressurization^103–106^, mechanical bending^107–112^, and electrostriction^113^, have been utilized to induce physical strain, controlling strain at the nanoscale space and exploring optical properties with nanoscale spatial resolution remain challenges.

**Fig. 4:**
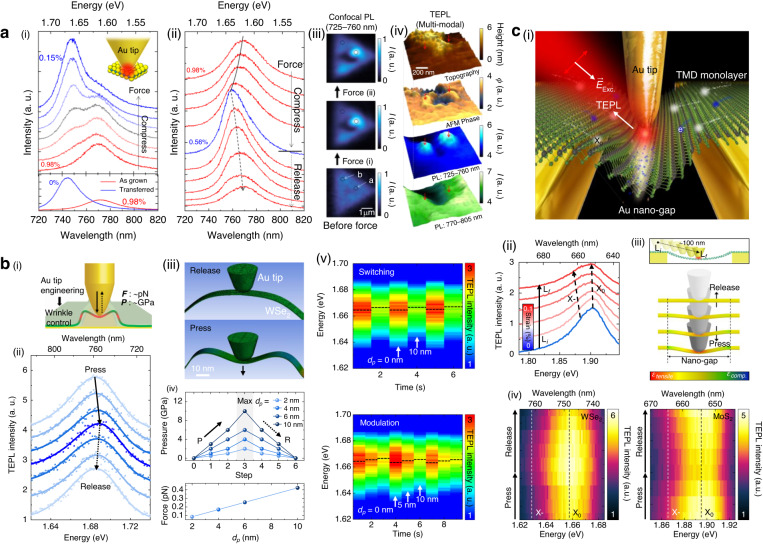
**Tip-induced strain engineering of TMD MLs via local pressure control. a** Modifications in TEPL spectra of the as-grown WSe_2_ monolayer with irreversible (i) and reversible (ii) changes of the strain on the crystal. (iii) Confocal PL images before and after releasing the irreversible (i) and reversible strain (ii). (iv) Nano-resolving of AFM topography, phase, blue shifted TEPL, and main TEPL for two strain released spots. **b** Strain engineering on the wrinkle structure in WSe_2_ monolayer. (i) Schematic of tip-induced strain control on a wrinkle structure. (ii) Reversibly changing TEPL spectra with the gradual press and release of the tip. (iii) A model representing the Au tip and the WSe_2_ wrinkle, designed to simulate the local pressure at the wrinkle apex caused by the tip. (iv) Computed local pressure and the force exerted on the wrinkle apex when the gold tip is pressed and released at various pressing depths ($d$_p_ = 2, 4, 6, and 10 nm). (v) Demonstration of switching and modulation of wrinkle TEPL by binary pressing depth and three discrete pressing depths. **c** Nanoscale strain engineering for exciton funneling and trion conversion. (i) Schematic of TMD monolayers transferred on an Au nanogap combined with TEPL spectroscopy. (ii) Simulation of strain distribution for pressured TMD monolayers on the nanogap with different tip positions. (iii) TEPL spectra of a MoS_2_ monolayer as the tip moves from the edge ($L$_i_) to the center ($L$_f_) of the nanogap. (iv) Reversible changes in TEPL spectra of WSe_2_ (left) and MoS_2_ (right) monolayers by modulating tip-induced pressure. Dashed lines demonstrate the energy of excitons (black) and trions (white). **a** Reproduced with permission^81^. Copyright [2016] American Chemical Society. **b** Reproduced with permission^28^. Copyright [2021] Wiley-VCH. **c** Reproduced with permission^29^. Copyright © 2022 Lee et al., some rights reserved; exclusive licensee American Association for the Advancement of Science. No claim to original U.S. Government Works. Distributed under a Creative Commons Attribution NonCommercial License 4.0 (CC BY-NC)

Recently, a promising approach called tip-induced strain engineering has facilitated the application of GPa-scale pressure to specific nanoscale regions while maintaining sub-diffraction-limited spatial resolution^114–122^. By considering the simple physical definition of pressure (force divided by area), a nanoscale tip can achieve GPa-scale pressure, despite its minute force at the pN–nN level. For instance, the changing TEPL responses of a WSe_2_ monolayer were demonstrated by releasing the intrinsic strain between the as-grown WSe_2_ monolayer and the substrate, as shown in Fig. 4a-(i)^81^. Similar strain-induced bandgap transition was observed for a WS_2_ monolayer^123^. By carefully applying local force and pressure using a plasmonic tip, the strain can be locally released in a reversible manner, resulting in the reversible changes in TEPL intensity and energy, as depicted in Fig. 4a-(ii). Moreover, confocal PL imaging before and after applying the tip-induced force and pressure revealed irreversible PL changes from the released strain, as illustrated in Fig. 4a-(iii). In addition, the nanoscale spatial resolution of TEPL spectroscopy provided detailed information about the indented region, as demonstrated in Fig. 4a-(iv). Notably, the TEPL image of the WSe_2_ flake near a nucleation site (NS) region displayed initially suppressed TEPL intensity, whereas the two pressed regions exhibited distinctly blueshifted and increased TEPL signals. These findings highlight the capability of nano-mechanical tip interactions to induce both reversible and irreversible release of local strain, offering a unique approach to control the PL energy and quantum yield of nanoscale defects in 2D materials.

In a recent study performed by Koo et al., the optical properties of nanoscale wrinkles in a WSe_2_ monolayer were dynamically controlled using GPa-scale pressure applied with a plasmonic tip, as depicted in Fig. 4b-(i)^28^. Figure 4b-(ii) illustrates the reversible modulation of PL intensity and energy as the tip gradually presses and releases the wrinkle structure. As the tip releases the intrinsic tensile strain on the wrinkle structure, the blueshifted PL energy is observed. At the wrinkle apex, the naturally-induced strain modifies the lattice structure, resulting in a reduced bandgap of the crystal. This reduced bandgap promotes exciton funneling into the wrinkle apex, leading to a higher PL quantum yield compared to that of the crystal face. Conversely, applying tip-pressure can release the strain at the wrinkle apex, which increases the bandgap energy and reduces exciton funneling, resulting in decreased PL intensity at the wrinkle apex. To accurately quantify the local pressure induced by the plasmonic Au tip on the WSe_2_ wrinkle, a numerical simulation was performed, and the results are shown in Fig. 4b-(iii). Remarkably, the study revealed that achieving GPa scale pressure requires an extremely low level of force. Moreover, this approach allowed for dynamic engineering of the radiative decay rate from nanoscale wrinkles through systematic vertical positioning of the plasmonic Au tip, as demonstrated in Fig. 4b-(iv). Through this concept, this study showcased the switching and modulation modes of wrinkle emission using both binary pressing depths and three discrete levels. These findings highlight the unique optical characteristics of the naturally-formed nanoscale wrinkles, which differentiate them from the optical properties of the crystal face. Consequently, this work signifies the potential of nanoscale wrinkles as nanoscale light-illuminating sources in 2D semiconductors.

Furthermore, in a recent study performed by Lee et al., the combination of the tip-induced strain engineering technique with a plasmonic nanogap structure enabled dynamic control of exciton funneling and trion conversion processes, as illustrated in Fig. 4c-(i)^29^. When WSe_2_ and MoS_2_ monolayers were transferred onto the nanogap, the naturally generated strain gradient resulting from the suspended geometry of the crystals led to a corresponding gradient of the bandgap, with energy minima at the nanogap center. Notably, this structure is an inverse of the naturally-formed wrinkles in 2D materials. Consequently, this strain gradient caused exciton funneling in a WSe_2_ monolayer and exciton-to-trion conversion in a MoS_2_ monolayer, as excess electrons in n-type semiconductors funneled together with neutral excitons. Figure 4c-(ii) displays the increased trion density at the bandgap minima, obtained through TEPL spectroscopy. By gradually pressing the suspended MoS_2_ monolayer on the nanogap structure, the maximum value of the strain gradient could be incrementally increased, as shown in Fig. 4c-(iii). As the tip pressed the suspended WSe_2_ monolayer, the increased tensile strain leads to the increased TEPL intensity of excitons without altering the TEPL intensity of trions, as depicted in Fig. 4c-(iv). Similar tip-induced strain effect exhibiting spectral shift in a WSe_2_ monolayer was also reported^124^, even though tip-enhanced nano-spectroscopy measurement was not performed. By contrast, the MoS_2_ monolayer exhibited a decrease in TEPL intensity of excitons along with an increase in TEPL intensity of trions, demonstrating exciton-to-trion conversion. Specifically, the gradual increase in pressure caused by the tip results in a progressive enhancement of the strain gradient, causing excitons and free electrons to be concentrated at the center of strain gradient. This increased concentration leads to the conversion of these confined excitons and free electrons into trions, ultimately resulting in a reduction in the intensity of neutral excitons, while the intensity of trions increases. In this work, the spatial positioning accuracy of ~0.2 nm and GPa-scale pressure of the plasmonic Au tip allowed for precise nanoscale control of exciton dynamics in a reversible manner, including the modulation of exciton funneling rate in a WSe_2_ monolayer and exciton-to-trion conversion rate in a MoS_2_ monolayer. These investigations revealed subtle excitonic behaviors in nanoscale regions, while the utilization of tip-induced control modality allowed for direct and reversible regulation of these excitonic phenomena. It is important to note that the materials subject to exploration and control extend beyond the 2D TMDs discussed in this study.

**Fig. 5:**
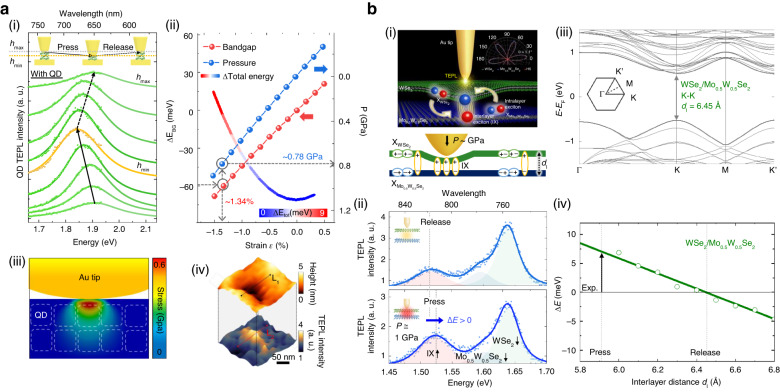
**Tip-induced bandgap engineering of quantum dot and 2D heterobilayer via local pressure control. a** Structural and electronic band deformation of pQD ensemble. (i) Reversible tip-pressure engineering of a single pQD. (ii) Bandgap energy shift and pressure as a function of applied strain along the c-axis. The free energy graph is represented separately in the background. (iii) Numerical simulation for the distribution of stress when the Au tip applies pressure on an ensemble of pQDs. (iv) AFM image of the pQD ensemble undergoing structural modifications due to tip-pressure (top) and corresponding TEPL intensity image (bottom). **b** Local modification of interlayer coupling strength. (i) Illustrations of tip-induced control of the interlayer coupling strength for a WSe_2_/Mo_0.5_W_0.5_Se_2_ heterobilayer. (ii) TEPL spectra of the WSe_2_/Mo_0.5_W_0.5_Se_2_ heterobilayer before (top) and after (bottom) applying the tip-pressure. (iii) Electronic band structure calculated for the equilibrium interlayer distance of $d$_I_ = 6.45 Å. (iv) Energy shift for K-K transition as a function of interlayer distance. **a** Reprinted with permission^125^. Copyright [2021] American Chemical Society. **b** Reproduced with permission^129^. Copyright [2023] Koo et al

The tip-induced pressure approach has opened up new avenues for investigating quantum light-matter interactions and tailoring them for various applications. In addition to nanoscale strain engineering of TMD monolayers, tip-induced strain engineering enables local modification of the lattice and electronic band structure of various low-dimensional quantum materials in a highly controlled manner. A recent study demonstrated the reversible tuning of emission energy of a single perovskite quantum dot (pQD) using GPa-scale pressure applied by a plasmonic Au tip^125^. By gradually pressing and releasing the single pQD, its emission properties can be precisely adjusted in real-time, as shown in the TEPL spectra in Fig. 5a-(i). Density functional theory (DFT) calculations quantified the applied strain of ~1.34% and the pressure of ~0.78 GPa required to achieve the experimentally obtained energy shift in PL spectra, as shown in Fig. 5a-(ii). In the case of the pQD ensemble, applying tip-pressure beyond a certain threshold leads to permanent modifications in their structural and radiative emitting properties, as depicted in Fig. 5a-(iii), (iv). The affected area in the ensemble exhibited structural deformation, accompanied by an increase in TEPL intensity. This alteration in PL intensity can be attributed to the reduced distance between neighboring pQDs, as they were compressed by the applied pressure. It decreased the plasmonic cavity mode volume between the tip and Au substrate, as shown in the correlation between the topography and TEPL images of Fig. 5a-(iii), (iv). The proposed tip-pressure engineering technique offered a unique approach to modulate the optical and electronic properties of perovskites at the single QD level. Furthermore, this study demonstrated that the plasmonic cavity formed underneath the plasmonic Au tip with the Au substrate suppresses the increased nonradiative decay rate from structural deformation, thereby preserving radiative properties even in the highly pressed regime. As another direction of applications, the ability to induce a phase transition by tip-pressure could have far-reaching implications in material sciences and the development of advanced nanoscale manipulation technologies for phase-transition materials, such as perovskite compounds^126^, topological insulators^127^, and high temperature superconductors^128^. It should be also noted that the demonstrated work confirmed the consistent results of the tip-pressure experiment when using different tips. This approach was also applied to TMD heterostructures to tune the PL energy of interlayer excitons. Figure 5b-(i) presented a conceptual illustration of nanoscale pressure engineering of a TMD heterobilayer using a plasmonic tip^129^. When the Au tip applies pressure onto a WSe_2_/Mo_0.5_W_0.5_Se_2_ heterobilayer, the interlayer distance becomes smaller, leading to an increase in interlayer coupling strength. Consequently, this effect leads to an increased PL intensity of interlayer excitons with a simultaneous decrease in the PL intensity of intralayer excitons, as shown in Fig. 5b-(ii). Furthermore, for local regions that already have a high interlayer coupling strength, the GPa-scale tip-pressure could induce bandgap and PL energy shifts in the heterobilayer due to modifications in the lattice structure. Figure 5b-(iii) exhibited DFT calculations showing an equilibrium interlayer distance of 6.45 Å for the untreated WSe_2_/Mo_0.5_W_0.5_Se_2_ heterobilayer. As demonstrated in Fig. 5b-(iv), reducing the interlayer distance resulted in an increased emission energy of the interlayer excitons. Therefore, the experimentally reduced interlayer distance could be estimated by monitoring the TEPL energy of interlayer excitons. Taking into account the distinctive characteristics of interlayer excitons, including extended valley polarization, coherence, and recombination times, this method establishes a foundation for local manipulation of interlayer excitons. This can potentially be extended to nano-integrated excitonic/trionic circuits and advanced optoelectronic devices in subsequent generations.

In this section, we have reviewed the recent developments and progress of the tip-induced pressure engineering approach applied to various low-dimensional quantum materials. For effective harnessing of nanoscale modifications in lattice structure, changes in bandgap and PL response, and a diverse range of quantum light-matter interactions, the ability to probe nanoscale optical phenomena is an indispensable prerequisite. When combined with TEPL spectroscopy, the demonstrated tip-induced GPa-pressure engineering approaches not only provide a comprehensive correlated analysis of the structural and optical properties of various materials but also offer a practical means for modifying mechanical and electronic properties at the desired nanoscale region. In addition, the effect of tip-induced pressure in the strong coupling regime has not been yet clarified due to the lack of suitable experimental approaches. We expect that the tip-induced GPa scale pressure-engineering technique could potentially open a pathway to investigate pressure-related optical phenomena in the strong coupling regime. It should be noted that since the Au tip is composed of a relatively soft metal, it is important to consider the potential structural modifications that may occur during tip-pressure experiments. In previous studies we have referenced, the experiments primarily focused on soft materials, such as nano-wrinkles in 2D materials, suspended 2D sheets on nanogaps, and perovskite QDs. This choice of materials aimed to minimize structural deformation of the Au tip. As a forward-looking perspective, we believe it would be beneficial to explore the fabrication of Au alloy tips with higher hardness. This approach has the potential to enhance the versatility and applicability of tip-pressure experiments, enabling investigations across a broader range of materials and systems.

### Tip-induced control of near-field polarization at the nanoscale

Polarization control of light in the far-field regime has been extensively investigated and applied for optical characterization and manipulation of light-matter interactions in photonic, plasmonic, and optoelectronic systems^130–138^. For instance, this approach can reveal the molecular or crystal orientation^139–141^, electronic band structures of low-dimensional semiconductors^142–145^, and light-matter coupling in hetero- and hybrid structures^146–149^. Likewise, polarization control in the near-field regime is also highly desired for studying nanoscale characteristics, i.e., coupled behavior of single quantum emitters^150–152^ and excitonic states in superlattice structures^153–155^. Yet, modulation of near-field polarization at the nanoscale remains challenging. Conventional tip-enhanced nano-spectroscopy is known to suffer from limited tunability of polarization at the tip apex since traditionally used plasmonic tips generate strong out-of-plane fields at the apex, while the corresponding in-plane fields are relatively weak^25^. Controlling optical field distribution at the tip apex can therefore increase sensitivity and enhance light-matter interaction with in-plane oriented dipole excitations, such as excitons in 2D materials^156^.

**Fig. 6:**
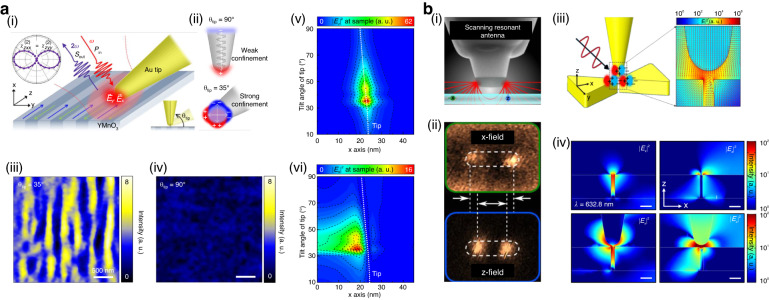
**Tip-induced nearfield polarization control by exploiting plasmonic cavity geometry. a** Near-field polarization control by tilting the conventional antenna tip. (i) Schematic of $x$-cut single-crystalline YMnO_3_ nano-crystallographic imaging using the tilted tip. (ii) Illustration of the geometric confinement for collective electron oscillations in conventional tip oriented at 90° (top) to the sample surface (top) and tilted tip oriented at 35° (bottom). Tip-enhanced SHG images measured with tips tilted at an angle of 35° (iii) and at 90° (iv). Calculations for the in-plane (v) and out-of-plane (vi) optical field intensities at the sample for varying tilt angle of the tip *(θ*_tip_). **b** Designed nanoantenna structures to manipulate the near-field polarization. (i) Schematic of scanning single molecule emitters using a special resonant dipole nanoantenna. (ii) Measured $x$- and $z$-field intensity from the selected individual molecules with $x$- and $z$-oriented dipole moments. (iii) Schematic of vector field polarization in a triple-tips nanostructure, which consists of a bowtie antenna structure and a tip (left), together with FDTD simulation result of plasmonic field enhancement in the triple-tips nanostructure (right). (iv) Calculated in-plane (|*E*_x_|^2^, left) and out-of-plane (|*E*_z_|^2^, right) field distributions of the bowtie structure without (top) and with (bottom) a vertically oriented tip. Reprinted with permission from ref. ^157^. Copyright [2018] American Chemical Society. **b**-(i), (ii) Reproduced with permission^162^. Copyright [2014] American Chemical Society. **b**-(iii), (iv) Reproduced with permission^165^. Copyright [2021] John Wiley and Sons

One approach to create substantial in-plane components in a conventional conical tip structure is by using a simple geometry modification, as shown in Fig. 6a-(i), (ii). Adjusting the angle of the tip axis with respect to the substrate under side illumination significantly increases the in-plane field component at the tip apex^157^, which is attributed to the geometry-dependent damping of the collective oscillations of electrons^158^. The electron oscillations are overdamped for a vertically aligned tip due to the semi-infinite geometry with only a single metal–dielectric interface, resulting in a weak localized surface plasmon resonance (LSPR). On the other hand, the collective electron oscillations for a tilted tip are confined within a finite volume near the apex, leading to an enhanced LSPR response for both the in-plane and out-of-plane components compared to the vertically aligned tip. The angle-dependent in-plane enhancement of the tilted tip is clearly demonstrated in the imaging of ferroelectric domains in single-crystalline YMnO_3_, as shown in Fig. 6a-(iii), (iv)^159,160^. When the tip is tilted at 35° to the sample plane, a distinct contrast between domains is observed in the tip-enhanced second harmonic generation (SHG) image, while the domains are barely noticeable when the tip is oriented vertically (*θ*_tip_ = 90°). To systematically evaluate the field enhancement of the tilted tip, the authors calculated nanoscale spatial distributions of the in-plane |*E*_x_|^2^ and out-of-plane |*E*_z_|^2^ components of the optical field for a variable tip tilting angle. The results shown in Fig. 6a-(v), (vi) demonstrate that both the in-plane and out-of-plane field components are maximized at the angle of 35°. Thus, through the experimental geometry of tip-enhanced nano-spectroscopy, one can substantially modify the near-field distribution and improve light-matter interactions between the tip and excitations in the studied sample.

Another approach to manipulating the near-field polarization in tip-enhanced nano-spectroscopy takes advantage of the geometry-dependent field distribution in plasmonic nano-antennas^161^. For example, the use of a resonant dipole antenna instead of a conventional tip has been demonstrated, as shown in Fig. 6b-(i)^33,162^. To produce the resonant antenna, a fiber tip was tapered using the heat pulling method, which is generally used for near-field scanning optical microscopy (NSOM), followed by Al deposition and focused ion beam (FIB) milling to fabricate a rectangular antenna with the dimensions of 200 nm × 60 nm. The fabricated rectangular structure provides spatially separated in-plane and out-of-plane field distributions in the tip-sample gap. Therefore, when raster scanned over a sample containing single molecules, the antenna structure allows extracting information on the dipole orientational of each molecule, as shown in Fig. 6b-(ii)^163,164^.

Instead of modifying the tip geometry, combining a conventional tip with additional plasmonic nanostructures has also been suggested as a way to modulate the near-field distribution. For example, a triple-sharp-tips structure has been proposed and demonstrated by integrating a bowtie antenna and a conventional tip, as shown in Fig. 6b-(iii)^165^. To study the spatial distribution of the in-plane and out-of-plane field components for such structure, optical field distribution has been calculated for a bowtie structure with and without the tip using finite-difference time-domain (FDTD) simulations, as shown in Fig. 6b-(iv). The results showed that the bowtie structure without the tip has a strong in-plane field component (|*E*_x_|^2^, top left panel) inside the gap while the corresponding out-of-plane field (|*E*_z_|^2^, top right panel) is significantly weaker. In contrast, the triple-sharp-tips structure formed in the presence of the tip exhibits a significant enhancement of both *E*_x_ and *E*_z_ fields at the bowtie cavity (bottom left and right panels, respectively). The authors further utilized the structure to induce and investigate localized excitons in a WSe_2_ monolayer. Due to the strain of the transferred WSe_2_ on the bowtie structure^166^ and strong field enhancement at the cavity, localized excitons at room temperature were observed at the cavity of the triple-sharp-tips structure^162^.

All approaches to control the near-field polarization reviewed in this section so far relied on modifying the geometry and structure of plasmonic tips. While these approaches in principle allowed one to manipulate the field distribution at the tip and enhance light-matter interaction for dipole moments oriented in-plane, which is difficult to achieve with conventional tip-enhanced nano-spectroscopy, the requirement for a special experimental configuration limits their potential applications. This calls for a more versatile method for controlling the near-field polarization.

**Fig. 7:**
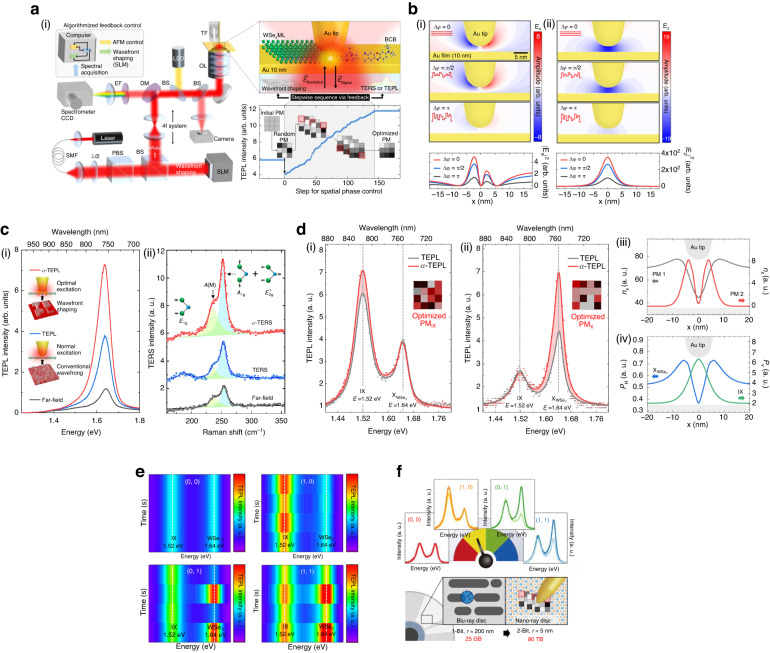
**Tip-induced nearfield polarization control by optical phase modulation. a** Dynamic wavefront shaping method with tip-enhanced nano-spectroscopy. (i) Schematic diagram of the experimental setup for adaptive tip-enhanced nano-spectroscopy. A He-Ne laser with a wavelength of 632.8 nm is spatially filtered and expanded to fully illuminate the active area of a spatial light modulator (SLM) for wavefront shaping. The wavefront-shaped beam is then imaged onto the back aperture of an objective lens (OL) within a 4f system, enabling dynamic manipulation of LSPR at the tip. (ii) TEPL intensity of a WSe_2_ monolayer changes as the phase mask (PM) is optimized using a stepwise sequential algorithm. The TEPL response with the optimal phase mask shows stronger enhancement than the conventional TEPL setup. **b** Simulated optical field distributions at the tip-sample gap for different phase masks. (i), (ii) In-plane (|*E*_x_|^2^) and out-of-plane (|*E*_z_|^2^) components of the optical field with respect to spatial phase variation. Intensity profiles of the in-plane (|*E*_x_|^2^) and out-of-plane (|*E*_z_|^2^) optical field at a horizontal plane 1 nm below the tip (bottom). **c** (i), (ii) Far-field PL/Raman spectrum (black) and TEPL/TERS spectra of a WSe_2_ monolayer without SLM (blue) and with optimal wavefront conditions using SLM (*a*-TEPL, red). **d** Selective TEPL modulation of intra- and interlayer exciton emission in a WSe_2_/Mo_0.5_W_0.5_Se_2_ heterobilayer via wavefront shaping. (i) Comparison of normal TEPL and *a*-TEPL spectra without (gray) and with (red) the optimized phase mask for the IX peak (PM_IX_). (ii) Comparison between normal TEPL and *a*-TEPL spectra without (gray) and with (red) the optimized phase mask for the ${{\rm{X}}}_{{{\rm{WSe}}}_{2}}$ peak (PM_X_). (iii) Simulated exciton density (*n*_x_) profiles of WSe_2_ intralayer excitons (${{\rm{X}}}_{{{\rm{WSe}}}_{2}}$) along the $x$-axis for two different model phase masks (PM1 and PM2). (iv) Simulated Purcell factors for the in-plane (${{\rm{X}}}_{{{\rm{WSe}}}_{2}}$, *P*_H_) and out-of-plane (IX, *P*_V_) dipoles as functions of the dipole position with respect to the tip. **e** Demonstration of optical switching in *a* nano-excitonic transistor using *a*-TEPL spectra of IX and ${{\rm{X}}}_{{{\rm{WSe}}}_{2}}$. These figures demonstrated data units of (0, 0), (1, 0), (0, 1), and (1, 1) for the operation of a 2-bit nano-excitonic transistor. **f** Representation of two-optical bit processing using the four distinct data units from (**e**) and comparison of the nano-ray disc utilizing a 2-bit nano-excitonic transistor, which has significantly higher data density and capacity, with a traditional Blu-ray disc. **a** Reproduced with permission^167^. Copyright [2021] Lee. **b** Reproduced with permission^167^. Copyright [2021] Lee. **c** Reproduced with permission^167^. Copyright [2021] Lee. **d** Reproduced with permission^168^. Copyright [2023] American Chemical Society. **e** Reproduced with permission^168^. Copyright [2023] American Chemical Society. **f** Reproduced with permission^168^. Copyright [2023] American Chemical Society

To address the aforementioned issue, optically controlled adaptive tip-enhanced nano-spectroscopy was demonstrated using wavefront shaping method, as shown in Fig. 7^167,168^. A spatial light modulator (SLM) integrated into a conventional tip-enhanced nano-spectroscopy setup allows manipulating the phase of incident light by dividing the laser beam into multiple segments and controlling the phase of each segment^169–171^. The SLM-shaped wavefront is then used to illuminate the tip-sample gap, and the target signal of the detected TEPL response is optimized using the sequential feedback algorithm, as shown in Fig. 7a-(ii). To understand the effect of SLM on the near-field polarization, the phase-dependent field enhancement at the tip is evaluated theoretically via FDTD simulations, with the results shown in Fig. 7b-(i), (ii). To examine the local field enhancement with respect to spatial phase difference, the incident light source was constructed with many optical sources, all with the same linear polarization but varying phase delays ranging from 0 to *π*. Both in-plane (1) and out-of-plane (2) near-field components were found to be sensitive to phase delay, which indicates that adjusting the phase delay provides accurate control on the local field enhancement and associated light-matter interactions. The calculated phase-dependent near-field polarization behavior was confirmed experimentally via measuring excitonic TEPL signals from a WSe_2_ monolayer sample. As depicted in Fig. 7c-(i), the PL intensity generated with a radially polarized excitation beam in the monolayer (black) is first considerably increased as the tip approached the sample (TEPL, blue), which is attributed to the conventional tip enhancement effect. The TEPL intensity is further enhanced by the adaptive optimization of the excitation wavefront with SLM (*a*-TEPL, red), more than twice compared to the conventional TEPL intensity, with the corresponding enhancement factor of ~4.4 × 10^4^, which is attributed to the stronger in-plane enhancement at the tip.

In addition, adaptive TERS (*a*-TERS) experiment was carried out on the same sample to analyze the effects of wavefront shaping on Raman responses as the vibrational modes have different orientation and enhancement mechanism. Figure 7c-(ii) shows the far-field Raman (black) and TERS spectra of a WSe_2_ monolayer, both with (red) and without (blue) wavefront shaping. The *a*-TERS intensities of *A*(M) (asymmetric phonon mode at the M point) and ${A}_{1g}+{E}_{2g}^{1}$ (out-of-plane vibration of Se atoms and in-plane vibration of W and Se atoms) modes were enhanced more than two times in comparison to the TERS signals in the absence of wavefront shaping, while the ${E}_{1g}$ (in-plane vibration of Se atoms) mode was slightly decreased. This result indicates that the phase mask optimization procedure enhances the vibrational modes in the out-of-plane direction $({A}_{1g})$, but not the in-plane modes $({E}_{1g})$ ^172,173^. The authors also point out that the additional enhancement by the wavefront shaping varies between different tips (1.3 ~ 2.5 times compared to normal TERS/TEPL), which is attribute to the nanoscale differences in the tip apex geometry. If the fabrication of identical tip shapes is feasible, it will enable the quantitative analysis of enhancement and field distribution.

In addition to the orientation-dependent enhancement of vibrational modes in *a*-TERS, selective enhancement of PL spectra for excitons with different dipole orientations has been demonstrated in a WSe_2_/Mo_0.5_W_0.5_Se_2_ heterostructure, as shown in Fig. 7d-(i), (ii)^168^. In the heterostructure, *a*-TEPL can selectively enhance intralayer excitons ${{\rm{X}}}_{{{\rm{WSe}}}_{2}}$ (in-plane dipole orientation) and interlayer excitons IX (out-of-plane dipole orientation) using different phase masks. The mechanism behind the selective control of two PL peaks relies on two aspects, namely, the different spatial distribution of excitons created for different phase masks and distinctive dependencies of radiative emission on in-plane coordinate for intra- and inter-layer excitons. As shown in Fig. 7d-(iii), the simulated spatial distributions of the exciton density are qualitatively different for two model phase masks corresponding to the in-plane (PM1, gray) and out-of-plane (PM2, red) polarized incident light. In addition, the Purcell factors calculated for horizontally (*P*_H_) and vertically (*P*_V_) oriented dipoles exhibit distinctly different dependencies on the in-plane exciton coordinate, corresponding to different radiative emission rates of ${{\rm{X}}}_{{{\rm{WSe}}}_{2}}$ and IX, as shown in Fig. 7d-(iv). The total *a*-TEPL response is then evaluated as a convolution of the spatial dependencies for the exciton density and Purcell factor, which yields selective enhancement of X or IX peaks for different phase mask, in agreement with the experimental results. Based on the demonstrated selective modulation of X and IX PL peaks, the authors proposed a conceptual device, an optically controlled nano-excitonic transistor. As shown in Fig. 7e, the proposed device can controllably generate two optical bits of data at the nanoscale. A 2-ternary digit (trit) system can be also developed based on the same idea, where the *a*-TEPL intensities of ${{\rm{X}}}_{{{\rm{WSe}}}_{2}}$ and IX can be controlled to represent −1, 0, and 1 digits. Furthermore, since the demonstrated approach has few-nm spatial resolution, it allows developing data storage and processing elements with data capacity exceeding that of a Blu-ray disc by more than 1000 times, as depicted in Fig. 7f. In addition, we envision that the near-field polarization control at the tip can further expand to light-matter interactions in the ultrafast time domain^174,175^. Notably, fs-pulsed laser excitation at plasmonic tips has been shown to reveal the nanoscale nonlinear optical phenomena in 2D van der Waals materials^176,177^. Furthermore, recent advancements in ultrafast spatiotemporal light control, utilizing active metasurfaces^178^ and SLM^179^, enable ultrafast wavefront manipulation with potential applications in near-field polarization control at the tip in both time and space domains.

In summary, this section provides an overview of the methods to control near-field polarization for enhancing tip-induced light-matter interactions. Strong in-plane enhancement can be enabled by modifying the geometry and structure of the tip, i.e., tilting a conventional tip, fabricating a resonant antenna at the tip end, and integrating a conventional tip with additional nanostructures. Adaptive wavefront shaping using SLM is suggested as a more versatile approach that enables selective enhancement and suppression of the desired optical responses in a conventional tip-enhanced nano-spectroscopy scheme without the need for modification of the tip structure. Given that this technique is still in its nascent stages, there is a pressing need for more comprehensive and systematic experiments aimed at characterizing the near-field polarization induced by wavefront shaping. These dedicated efforts are poised to advance our comprehension of adaptive near-field optics, paving the way for a multitude of potential applications and expanded capabilities. The ability to control near-field polarization introduces a new degree of freedom for dynamic all-optical control of low-dimensional quantum materials, which will facilitate the development of novel compact photonic and optoelectronic devices for processing classical and quantum information.

## Conclusions

In this comprehensive review, we have delved into the recent advancements in tip-induced control of quantum light-matter interactions at the nanoscale, with a particular focus on its applications in low-dimensional quantum materials. Going beyond the conventional uses of SPM^180,181^ and tip-enhanced nano-spectroscopy^20^ to overcome optical diffraction limits and improve sensitivity, we explored how incorporating newly developed control functions enables the manipulation of tip-enhanced spectroscopy to precisely influence light-matter interactions at the nanoscale.

Throughout the review, we highlighted three unique approaches for controlling nanoscale light-matter interactions. Firstly, we discussed the tip-induced control of radiative emission via cavity-gap regulation. By carefully adjusting the distance between the tip and the sample with sub-nanometer precision in ambient conditions, researchers demonstrated that the mode volume of the tip-cavity can be dynamically engineered, allowing us to control the radiative decay rate of emitters positioned within the cavity. The controllable tip-cavity mode volume significantly changes the field enhancement and Purcell factor, which in turn modifies the radiative emitting properties of low-dimensional quantum materials, such as dark exciton emission^25,182,183^ and the coupling strength between the cavity and emitters^31,33,184,185^.

Secondly, we explored the emerging field of tip-induced control of excitonic behaviors through GPa-scale pressure engineering. In addition to traditional tip indentation methods^186,187^, we showed how combining this approach with tip-enhanced nano-spectroscopy allows us to modify the mechanical and electronic properties of specific nanoscale regions while investigating the structural and optical properties of low-dimensional quantum materials, such as TMD mono- and bi-layers and perovskite QDs.

Lastly, we examined the promising avenue of tip-induced near-field polarization controls and their wide-ranging applications. Traditionally, physical modifications to the tip, such as the tilted-tip method and the fabrication of plasmonic structures, were employed to change the direction of near-field polarization. However, recent advances in adaptive optics techniques have provided more versatile and generalizable approaches, even with normal plasmonic tips. By reconstructing the wavefront of the excitation beam using SLM, researchers demonstrated the ability to achieve arbitrary near-field polarization states at the tip apex. This all-optical near-field polarization control technique selectively enhances or suppresses quantum light-matter interactions, opening up possibilities for various optoelectronic device applications.

In addition to the demonstrated tip-induced control approaches, numerous other methods can be combined with tip-enhanced nano-spectroscopy. For instance, magnetic field or force from magnetized tips can manipulate light-matter interactions by tuning the magneto-optical resonance for various systems^188,189^. Likewise, conductive tips can provide an additional modulation parameter through the local electric field, influencing plasmonic and electronic systems^190,191^. Furthermore, different approaches can be employed together to manipulate tip-induced light-matter interactions in a hybrid manner, for example, tip-induced hot electron injection into a TMD heterobilayer with modifications to the tip-cavity gap^126^.

In this review, while our discussions mainly focused on the optical phenomena in the visible and near-infrared (NIR) range, the tip-induced control approaches can also be applied to ultraviolet (UV)^192,193^, infrared (IR)^194,195^, and even Terahertz range^196,197^. Additionally, the tip-induced control approaches can be extended to chemical and biological systems, allowing manipulation of molecular dynamics at the single-molecule level^43,198^ and influencing photosynthesis in biological materials^199,200^.

In conclusion, this review showcases the exciting progress in tip-induced control of quantum light-matter interactions at the nanoscale, particularly in the realm of low-dimensional quantum materials. These novel approaches, including cavity-gap control, GPa-scale pressure engineering, and all-optical near-field polarization control, offer promising opportunities for tailoring and manipulating light-matter interactions in nanoscale systems. The insights presented here contribute to advancing the field of nanophotonics and lay the foundation for the development of cutting-edge optoelectronic devices with unprecedented capabilities.
